# Complete Genome Sequence of *Photobacterium* sp. Strain J15, Isolated from Seawater of Southwestern Johor, Malaysia

**DOI:** 10.1128/genomeA.00739-16

**Published:** 2016-07-28

**Authors:** Noordiyanah Nadhirah Roslan, Suriana Sabri, Siti Nurbaya Oslan, Syarul Nataqain Baharum, Thean Chor Leow

**Affiliations:** a Enzyme and Microbial Technology Research Center, Faculty of Biotechnology and Biomolecular Sciences, Universiti Putra Malaysia, Serdang, Selangor, Malaysia; b Department of Microbiology, Faculty of Biotechnology and Biomolecular Sciences, Universiti Putra Malaysia, Serdang, Selangor, Malaysia; c Department of Biochemistry, Faculty of Biotechnology and Biomolecular Sciences, Universiti Putra Malaysia, Serdang, Selangor, Malaysia; d Institute of Systems Biology (INBIOSIS), Universiti Kebangsaan Malaysia, Bangi, Selangor, Malaysia; e Department of Cell and Molecular Biology, Faculty of Biotechnology and Biomolecular Sciences, Universiti Putra Malaysia, Serdang, Selangor, Malaysia

## Abstract

Here, we report the genome sequences of *Photobacterium* sp. strain J15, isolated from seawater in Johor, Malaysia, with the ability to produce lipase and asparaginase. The PacBio genome sequence analysis of *Photobacterium* sp. strain J15 generated revealed its potential in producing enzymes with different catalytic functions.

## GENOME ANNOUNCEMENT

The genus *Photobacterium* was one of the earliest bacterial taxa known for its bioluminescence properties and could be found easily in marine habitat (Beijerinck 1889) (1). The study of the *Photobacterium* lags behind its close relatives *Aliivibrio* and *Vibrio* (2), which makes it quite hard to infer the precise evolutionary study of this genus.

*Photobacterium* sp. strain J15 (UPMC 398, DSM 25402) is a Gram-negative bacterium isolated from seawater in Tanjung Pelepas, Johor, Malaysia. It was initially studied for its lipase- and asparaginase-producing properties, both of which are important in the food and pharmaceutical industries (3, 4). The draft genome of *Photobacterium* sp. strain J15 was determined and annotated in order to gain further insight into the properties of the bacterium, including the metabolism pathways of industrial interest.

Whole-genome sequencing was carried out using the PacBio RS II system, generating one SMRT cell of sequencing data using P6-C4 chemistry. Total throughput of one SMRT cell with a 20-kb insert library amounted to 1,008,255,806 bp of data after quality filtering. After preprocessing, 215,204 reads were assembled by the PacBio Hierarchical Genome Assembly Process version 2.0 (HGAP 2.0) in SMRT Portal version 2.1.1.

The final assembly comprised three contigs with a total size of 5,684,538 nucleotides and a GC content of 46.39%. Next, an annotation was performed using the generated scaffolds. A total of 4,924 protein-coding sequences (≥33 amino acids) were predicted by Prodigal version 2.6, and functions of 4,811 (97.7%) genes of *Photobacterium* sp. strain J15 were predicted. KEGG metabolic pathway mappings were also carried out, resulting in 874 proteins with a KEGG assignment. A total of 180 tRNAs and 40 rRNAs (14 copies of 5S, 13 copies of 16S, and 13 copies of 23S) were identified by tRNAscan version 1.23 and rRNAmmer version 1.2, respectively.

In addition to the lipase, protease, and asparaginase genes found in the genome sequence (5, 6), genes encoding the arsenic-resistance protein Acr3 were found, which suggests the ability of this strain to confer resistance from arsenic. Furthermore, the genes encoding for multidrug resistance, chloramphenicol-resistance proteins, cobalt-zinc-cadmium resistance proteins, and tellurite resistance proteins were also found. The parkinsonism-associated protein DJ-1, which was recently determined to have antiglycation and antiaging properties (7), was also found in the genome sequence of this bacterium.

The information on the evolution of *Photobacterium* genomes is very limited. Therefore, the complete genome of *Photobacterium* sp. strain J15 will allow for comparative genomics study to unravel the evolution of *Photobacterium* spp.

### Nucleotide sequence accession numbers.

This whole-genome shotgun project has been deposited at DDBJ/ENA/GenBank under the accession number LSNH00000000. The version described in this paper is the first version, LSNH01000000.
